# A new recurring chromosome 13 abnormality in two older patients with *de novo* acute myeloid leukemia: An Indian experience

**DOI:** 10.4103/0971-6866.60190

**Published:** 2009

**Authors:** P. J. Trivedi, P. S. Patel, M. M. Brahmbhatt, B. P. Patel, S. B. Gajjar, E. N. Dalal, S. N. Shukla, P. M. Shah, S. R. Bakshi

**Affiliations:** Department of Cell Biology Division, The Gujarat Cancer and Research Institute, NCH Campus, Asarwa, Ahmedabad - 380 016, India; 1 Department of Biochemistry Research Division, The Gujarat Cancer and Research Institute, NCH Campus, Asarwa, Ahmedabad - 380 016, India; 2 Department of Medical Oncology, The Gujarat Cancer and Research Institute, NCH Campus, Asarwa, Ahmedabad - 380 016, India

**Keywords:** Acute myeloid leukemia-M1, recurrent, sole abnormality, trisomy 13

## Abstract

We report here two cases of trisomy 13 in acute myeloid leukemia M1 subtype. short-term unstimulated bone marrow and peripheral blood lymphocyte culture showed 47, XY, +13 in all metaphase plates and trisomy 13 was confirmed with whole chromosome paint probes. Trisomy 13 in AML-M1 is a rare numerical abnormality. This is the first Indian report of sole trisomy 13 in AML-M1. Here, we present two cases of elder male patients, which may constitute a distinct subtype.

## Introduction

Trisomy 13 is a rare recurring clonal chromosomal aberration with an incidence of 2.4% in *de novo* acute myeloid leukemia (AML).[1] Tetrasomy 13 can occur in different cases of acute leukemia with trisomy 13 as the primary cytogenetic abnormality or can be associated with additional abnormalities following transformation. Only four cases of primary acquired isolated tetrasomy have been described in patients with undifferentiated AML. The possibility that isolated tetrasomy 13 may represent an independent poor prognostic factor could be suggested by the poor outcome therapy in patients and in previously reported cases. However, responses to intensive chemotherapy in older patients (below 60 years of age) are lower than with younger patients, and all described cases of isolated tetrasomy 13 occurred in elderly subjects.[2]

## Materials and Methods

### Case 1

A 75 years old male with chief complaints of cough and breathlessness was referred to the Institute in August 2006. He had a past history of smoking (bidi 10/day/10 years). The bone marrow (BM) aspirate impression showed hypercellular marrow with marked proliferation of tumor blast population (>90%).The blasts were medium to large in size, with fine chromatin, two to five nucleoli and scanty bluish cytoplasm. The M:E ratio was altered. Occasional megakaryocytes were seen, which were suggestive of AML-M1. The peripheral blood lymphocyte culture (PBLC) report revealed hemoglobin 7.4 g/dL, white blood cell (WBC) count 82.7 × 10^3^/cm and platelet count 60 × 10^9^/UL.

### Case 2

A 63 years old male patient with complaints of fever, weakness and symptoms of anemia visited the Institute in April 2007. The BM report showed normal marrow component replaced by blast cells (75%). Cells are medium to large in size, having zero to two nucleoli and scanty bluish cytoplasm. Few erythroid precursors were seen with normoblastic erythropoiesis. The M:E ratio was altered and megakaryocytes were not seen, which was suggestive of AML-M1. The PBLC report showed hemoglobin 6.7 g/dL, WBC count 65.3 × 10^3^/ cm, platelet count 35 × 10^3^/UL, blast 88%, PAS-ve and Sudan block B +ve.

### Chromosome preparation

A G-banded chromosome study was performed using the standard cytogenetic protocol. Briefly, unstimulated cultures of BM aspirate were set-up in RPMI-1640 medium supplemented with 20% newborn calf serum, L-glutamine and antibiotics (penicillin and streptomycin). The cells were cultured for 24 and 48 h in a 5% CO_2_ incubator. Following overnight incubation in the presence of Colcemid (10 μl/8 ml of culture), the cultures were exposed to hypotonic solution (0.075 mol/L KCl) and fixed with methanol: acetic acid (3:1). The slides were prepared by the air dry method and stained with GTG banding. Twenty metaphases were analyzed and karyograms were prepared using the Cytovision computer-assisted karyotyping system (Applied Imaging, Newcastle Upon Tyne, UK). The karyotypes were described according to the International System for Human Cytogenetics Nomenclature.[3]

### Fluorescent in situ hybridization preparation

FISH was performed on interphase and metaphase cells following the manufacturer's (Abbott Molecular Inc., Des Plaines, IL, USA) guidelines. Whole chromosome paint for chromosome 13 with spectrum green was applied to confirm trisomy or cryptic rearrangements of chromosome 13.

## Results

### Chromosome analysis

**Figure 1:**
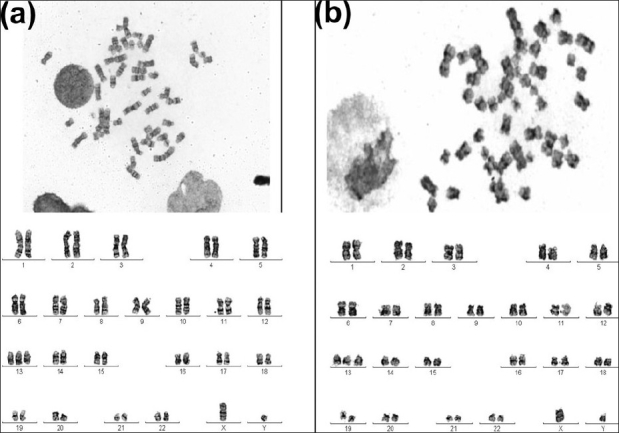
Representative GTG-banded karyotypes depicting trisomy 13 as the sole anomaly in bone marrow cells of (a) case 1-75/M and (b) case 2-63/M

Classical chromosome analysis showed an abnormal male chromosome complement in both the cases. In case 1, all the metaphases were 47, XY, +13[15], whereas case 2 showed 46, XY[10]/47, XY, +13[5] [Figure 1a and b]

### Fluorescent in situ hybridization analysis

**Figure 2:**
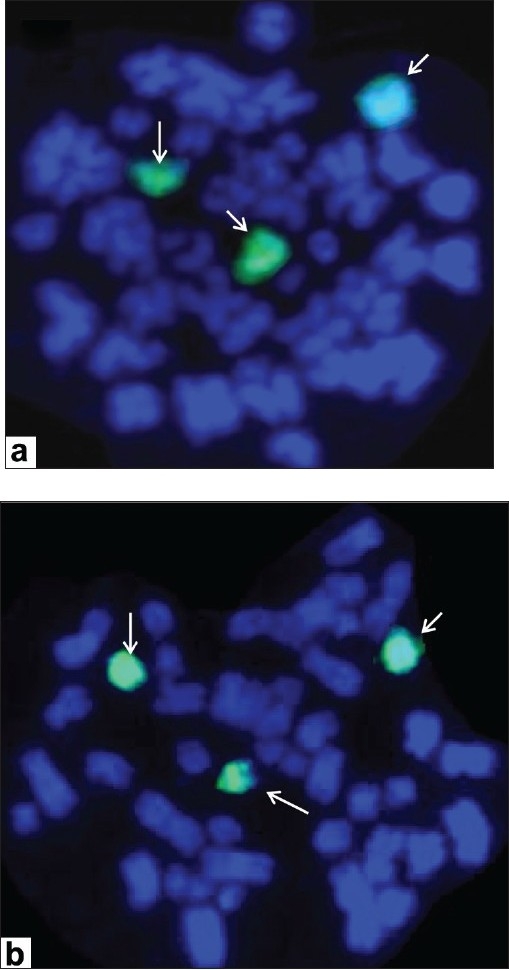
Representative metaphases following fluorescent *in situ* hybridization with spectrum GreenTM-labeled whole chromosome paint probe for chromosome 13 depicting trisomy 13 (arrows) as the sole anomaly in bone marrow cells of (a) case 1 and (b) case 2

The whole chromosome paint FISH for chromo some 13 showed three green signals, which confirmed trisomy of chromosome 13 with no other cryptic rearrangements in both the cases. Case 2 showed mixed clone, i.e. two and three signals for chromo some 13 [Figure 2a and b].

## Discussion

Deregulated function of FLT1 and Rb1 genes situated on chromosome 13 might be involved in the development of transformation of undifferentiated myeloid cells.[4] Retrospective and prospective studies have shown that specific chromosome abnormalities are of significant prognostic value. Abnormalities such as inv(16), t(15;17), and t(8;21) in myeloid leukemia have been associated with longer survivals, whereas monosomy 7, 11q23 rearrangements and the Philadelphia chromosome are predictors of a poor outcome. Large prospective clinical trials are necessary to assess the prognostic significance of other, less common recurring chromosome abnormalities in acute leukemia. The identification of risk groups based on specific chromosomal findings will have major clinical implications, especially with regard to the design of more risk-specific treatment protocols. In addition, the finding of new recurring chromosome abnormalities will provide insight into the gene region(s) that may be important in malignant transformation. A similar propensity for morphologic and immunologic lineage heterogeneity, as observed in trisomy 13, has been demonstrated for other recurring chromosome abnormalities, the Philadelphia chromosome and 11q23 abnormalities being classic examples. Within the myeloid series, lineage heterogeneity has been shown for leukemia, exhibiting trisomy 8 or monosomy 7.[5]

Baer *et al*. reported that +13 was observed in older male patients with the average age of 64 years, including 29 males and 7 females. Both the patients in the present study, were old-aged (60 and 68 years) males. It has also been suggested that trisomy 13 is a poor prognostic factor in acute leukemia. Reports suggested that short terms of complete remission were generally seen in these kinds of patients. It has been reported that patients with platelet counts ≤100 × 10^9^/L survive longer as compared with patients with platelet counts of ≥100 × 10^9^/L; the platelet count of patient 2 on admission was 35 × 10^9^/L. It has also been suggested that trisomy 13 is frequently seen in truck drivers, heavy equipment operators or cigarette smokers.[6] Case 1 was an electrician and a chronic smoker and expired within 1 week of diagnosis. Case 2 was lost to follow-up. The features of Indian patients in acute leukemia with trisomy 13 are not known. To the best of our knowledge, no case with trisomy 13 as a sole abnormality has been reported earlier in India.[7] More cases will be necessary to understand the correlation of trisomy 13 in neoplastic progression.
